# Translational compensation of genomic instability in neuroblastoma

**DOI:** 10.1038/srep14364

**Published:** 2015-09-24

**Authors:** Erik Dassi, Valentina Greco, Viktoryia Sidarovich, Paola Zuccotti, Natalia Arseni, Paola Scaruffi, Gian Paolo Tonini, Alessandro Quattrone

**Affiliations:** 1 Laboratory of Translational Genomics, Centre for Integrative Biology, University of Trento, Italy; 2 Center of Physiopathology of Human Reproduction, Unit of Obstetrics and Gynecology, IRCSS A.O.U. San Martino IST, Genova, Italy; 3 Neuroblastoma Laboratory, Pediatric Research Institute, Fondazione Città della Speranza, Padova, Italy

## Abstract

Cancer-associated gene expression imbalances are conventionally studied at the genomic, epigenomic and transcriptomic levels. Given the relevance of translational control in determining cell phenotypes, we evaluated the translatome, i.e., the transcriptome engaged in translation, as a descriptor of the effects of genetic instability in cancer. We performed this evaluation in high-risk neuroblastomas, which are characterized by a low frequency of point mutations or known cancer-driving genes and by the presence of several segmental chromosomal aberrations that produce gene-copy imbalances that guide aggressiveness. We thus integrated genome, transcriptome, translatome and miRome profiles in a representative panel of high-risk neuroblastoma cell lines. We identified a number of genes whose genomic imbalance was corrected by compensatory adaptations in translational efficiency. The transcriptomic level of these genes was predictive of poor prognosis in more than half of cases, and the genomic imbalances found in their loci were shared by 27 other tumor types. This homeostatic process is also not limited to copy number-altered genes, as we showed the translational stoichiometric rebalance of histone genes. We suggest that the translational buffering of fluctuations in these dose-sensitive transcripts is a potential driving process of neuroblastoma evolution.

Arising predominantly in the first two years of life, neuroblastoma is the most common cancer in infancy^1^. This cancer develops from the neural crest cells of the sympathetic nervous system and is classified as either aggressive or benign, with the latter most often encountering spontaneous regression (stage 4S) or gradual maturation to ganglioneuroma^2^,^3^. Aggressive neuroblastomas are further classified based on the presence of the MYCN amplification (appearing in ~16% of patients and associated with the worst prognosis of all subtypes^2^) and segmental aberrations, such as the loss of chromosome arms 1p and 11q or the gain of chromosome arm 17q^2^. Patients with MYCN-amplified neuroblastoma and segmental aberrations have a particularly poor prognosis, with an overall 5-year survival rate of only 30%^1^.

Cancer genetic instability is most often studied at the genomic, epigenomic and transcriptomic levels, thus mainly focusing on the effects of genomic alterations on transcription and splicing. However, several recent works have shown that translational control is a powerful determinant of proteome variation and cell phenotypes^4^. In a landmark study, Schwanhäusser *et al.* demonstrated that, due to translational control, mRNA steady-state levels are a poor proxy for their corresponding protein levels^5^,^6^. Moreover, others and we have shown that variations in transcriptome profiles induced by various stimuli are profoundly reprogrammed at the translational level^7^,^8^,^9^. In cancer tissues, genomic lesions affecting translation factors, RNA-binding proteins (RBPs) and non-coding RNAs alter this physiological reshaping of gene expression by translational efficiency. These alterations can produce a derangement of the translation machinery, the downstream effects of which are not detectable by transcriptome profiling^10^,^11^.

Incorporating translational efficiency estimation into mRNA profiling would generate molecular portraits that are closer to actual protein levels, thus helping to reveal the involvement of translational control alterations in tumor onset and progression, as previously proposed^12^. Such information could be obtained by translatomic profiling, which consists of polysome isolation by sucrose-gradient separation^13^ and the evaluation of mRNA content by high-throughput methods. The use of this approach in tumor cell lines or mouse tissues has so far been limited to a few reports^10^,^11^,^12^ and, to the best of our knowledge, no translatomic study has been performed on human tumor samples.

We present here for the first time the integrative profiling of thirteen MYCN-amplified neuroblastoma cell lines at the genomic, transcriptomic and translatomic levels. By integrating these datasets, we describe the prevalence of a compensatory behavior, induced by translational control, over a set of genes affected by recurrent copy number alterations (CNAs). These genes are often associated with prognosis and subject to concordant genomic alterations in 27 other tumor types. Such compensatory behavior is not limited to imbalanced loci, as we report effects on protein complex-forming genes and specifically validate this behavior in histone genes. We thus report here a new mechanism by which neuroblastoma cells can overcome fitness disruptions caused by genomic rearrangements.

## Results

### Translational control alterations in neuroblastoma

**Figure 1:**
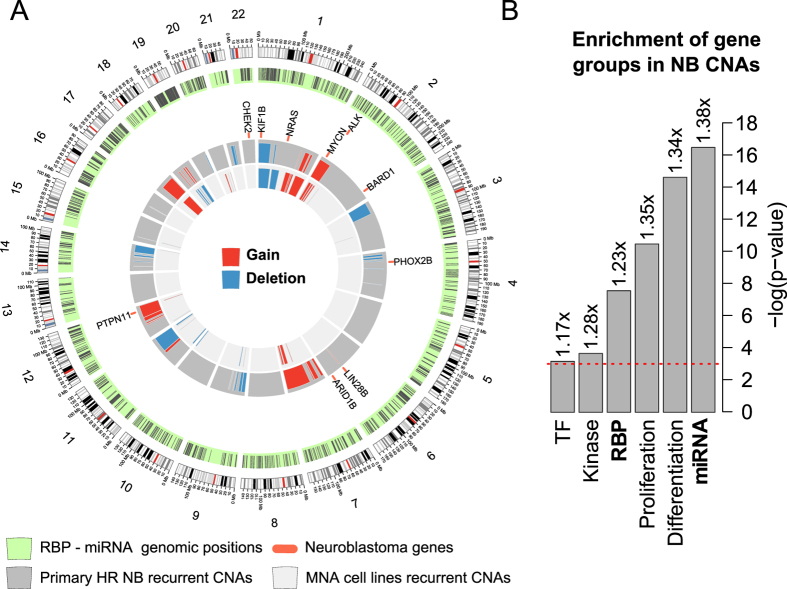
A map of neuroblastoma genomic alterations potentially affecting translation. (**A**) Recurrent copy number alterations in a set of 26 publicly available primary high-risk neuroblastoma samples and in our MYCN-amplified neuroblastoma cell lines dataset measured by aCGH analysis on the same platform. The outermost circle depicts chromosomes and related cytobands. Further in, the light green circle represents each RBP and miRNA gene with a black line at its corresponding genomic position. The dark grey circle represents recurrent alterations for the primary high-risk neuroblastomas, and the light grey circle displays the same for the MYCN-amplified neuroblastoma cell lines. Blue chunks represent deleted genome segments, and red chunks represent copy number gain/amplification events. The intermediate white circle indicates with an orange bar the genome position for the set of genes known to be involved in neuroblastoma onset and progression. (**B**) Enrichment p-values (Fisher’s test) for genes belonging to several categories and falling into a CNA in the set of MYCN-amplified neuroblastoma cell lines we profiled: RBPs, miRNAs, transcription factors (TF), kinases, and proliferation- and differentiation-related genes. The length of the bar represents the Benjamini-Hochberg-corrected enrichment p-value expressed as −log(p-value), thus showing higher values for lower p-values; the number on top of the bars represents the fold enrichment for the gene category in the CNA gene set over the genome, and the red dotted line indicates the significance threshold at 0.05.

We first sought to understand whether neuroblastoma genomic alterations could impact genes involved in post-transcriptional regulation. We considered 26 CNA profiles of high-risk neuroblastomas^14^ and analyzed their genomic structures. Because RBPs and miRNAs are the most documented trans-factors involved in post-transcriptional regulation, we reported their genomic distribution (Fig. 1A), observing a considerable proportion of them within altered regions in high-risk neuroblastomas. In particular, 490 RBP loci and 500 miRNA loci are altered (27.3% and 32.9%, as of miRBase 20^15^ of the total, respectively). Therefore, at least one out of four loci of genes involved in translational control is genomically imbalanced in high-risk neuroblastomas. Given the prevalence of MYCN-amplified tumors in the high-risk class, their relatively homogeneous genomic alteration profile (markedly different from that of non-MYCN-amplified tumors), their sheer aggressiveness^2^ and their unfavorable prognosis, we focused our analysis on this specific neuroblastoma subtype. We thus constructed an array of comparative genomic hybridization profiles for 13 primary (not sub-cloned *in vitro*) MYCN-amplified neuroblastoma cell lines and found them to be substantially superimposable with those of the tumors reported above (Fig. 1A); our cell-line sample is therefore representative of the genomic alteration pattern of high-risk neuroblastoma tumors. As for the neuroblastoma tumor profiles, many genes, listed in Supplementary Table S1, fall into CNA regions: 370 RBPs (20.7%) and 399 human miRNAs (22.8%) are indeed gained or lost in these cell lines. Comparing these figures with those of traditional cancer-enriched gene categories (kinases, transcription factors and genes implicated in proliferation and differentiation programs), we found that, as shown in Fig. 1B, RBPs and miRNAs are also significantly enriched in the CNA regions (Fisher’s test p = 5.13E-04 and 6.79E-08, respectively). Taken together, these data suggest that RBPs and miRNAs can be as important as traditional cancer-enriched gene categories in the evolution of neuroblastoma.

**Figure 2:**
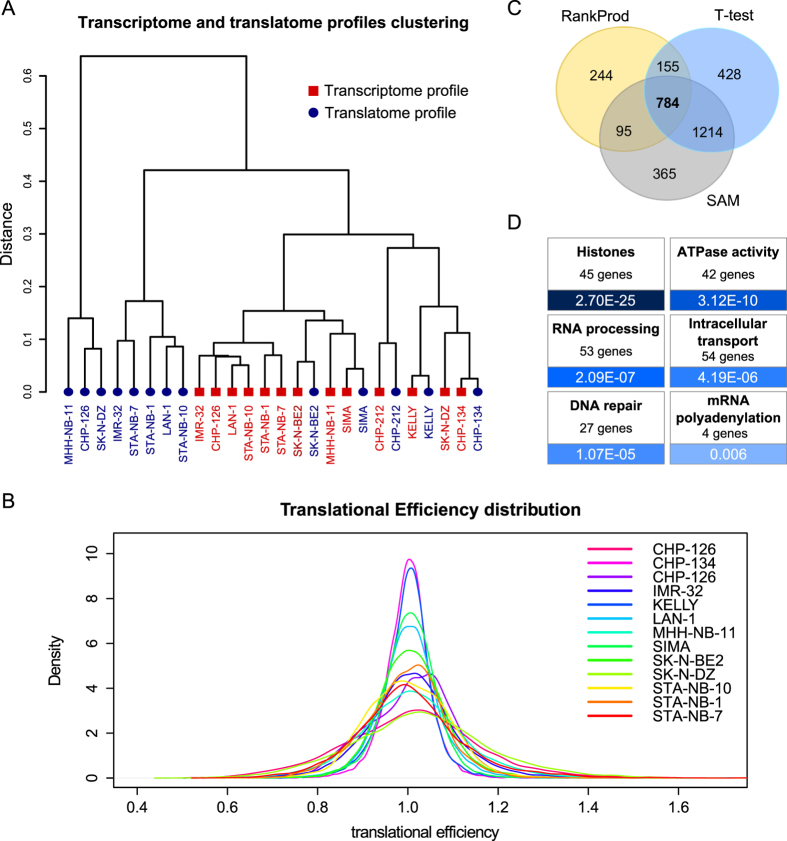
Evidence for widespread translational regulation in the MYCN-amplified neuroblastoma cell lines. (**A**) Hierarchical clustering of the transcriptome and translatome profiles for the MYCN-amplified neuroblastoma cell lines. Blue circles indicate translatomic profiles, and red squares indicate transcriptome profiles. (**B**) Translational efficiency distribution (computed as translatome/transcriptome for each gene) for the MYCN-amplified neuroblastoma cell lines that were profiled. Deviations from 1 represent the presence of translational control, either repressing (values < 1) or enhancing (values > 1). Tail size varies between samples but indicates a non-negligible degree of translational control for all the lines. (**C**) Venn diagram depicting the intersections of genes identified by the three methods as differentially abundant between the transcriptome and translatome profiles. (**D**) Functional themes that were found to be enriched in the differentially represented (in the transcriptome and the translatome) gene set. The blue gradient represents the corrected p-value from more significant (darker blue) to less significant (lighter blue).

Given the frequent alteration of these genes in MYCN-amplified neuroblastoma genomic profiles, we studied the available cell lines assuming that translational control has a role in the phenotype of this tumor. We thus profiled the total and polysomal mRNA and miRNA levels of these cells using a microarray platform. Surprisingly, the profile hierarchical clustering, as shown in Fig. 2A, indicated that for most cell lines (8/13, albeit in two distinct clusters), translatomic profiles cluster with other translatomic profiles, rather than with their respective transcriptome profiles. Furthermore, a PCA analysis and a k-means clustering (shown in Supplementary Fig. S1) also suggest the same pattern, with the latter approach identifying two clusters comprised of only translatomic profiles (including 11/13 of these) and a third cluster that included all transcriptome profiles and the remaining two translatomic profiles. Therefore, substantial translational control may be responsible for the considerable divergence between the two levels of mRNA analysis in each cell line, and this control may act similarly in different cell lines harboring similar genomic alterations.

To further investigate this phenomenon, we plotted the distribution of the mRNA translational efficiency (TE, defined as the ratio between translatomic and transcriptomic levels) for each cell line; deviations from 1 indicate translational repression (TE < 1) or translational enhancement (TE > 1). As shown in Fig. 2B, whereas the amount of control to which each cell line is subjected to varies, all of them have a considerable number of mRNAs with TE < or > 1, as represented by the fat distribution tails. Scatterplots of transcriptomic versus translatomic expression levels are also shown for all cell lines in Supplementary Figure S2.

By computing significant differences between the transcriptomic and translatomic signals, we then proceeded to identify mRNAs under consistently active translational control across the cell lines. We employed three algorithms: RankProd^16^, T-test and SAM^17^. As shown in Fig. 2C, all three identified a considerable number of mRNAs (11.7% for RankProd, 23.9% for T-test and 24.2% for SAM) as differentially abundant between levels, hinting again at the widespread engagement of translational regulation in these cell lines. The diagram shows a good agreement in the mRNAs predicted to be significantly regulated by all methods: the most conservative method, RankProd, shares 61.3% of its genes with T-test and SAM. The ranking approach adopted by RankProd abstracts from absolute signal means, thus alleviating potential background noise differences in the total and polysomal mRNA abundance data. This fact, combined with the higher robustness granted by the more conservative RankProd estimate of regulated mRNAs, which minimizes false-positive calls, eventually led us to use the RankProd results for subsequent analyses. These *differentially represented* genes were first functionally studied with an ontological enrichment analysis: as shown in Fig. 2D, the enriched themes include *histones* (up; i.e., more represented in the polysomal than in the total profiles), which is composed of 45 genes of all histone types (H1, H2A/B, H3 and H4), *ATPase activity* (42 down genes; i.e., less represented in the polysomal than in the total profiles), *RNA processing* (53 down genes), intracellular transport (54 down genes), *DNA repair* (27 down genes, including several SMC family members and chromatin-associated enzymes such as PARP1 and APEX1) and *mRNA polyadenylation* (PABPC1, CPSF1 and others, all down).

### Translational efficiency reprograms the gene expression of genomically altered genes

**Figure 3:**
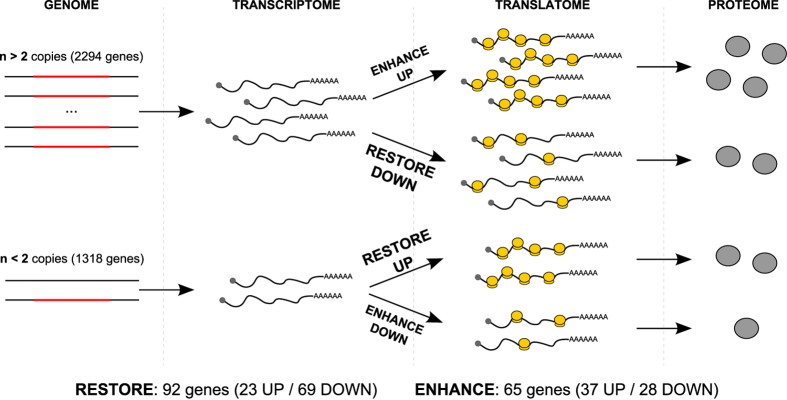
Translational compensatory and magnifying patterns for CNA-affected genes. The potential behaviors of genes that are under translational regulation in neuroblastoma and that are affected by collinear copy-number alterations are shown. We describe four possible situations: **ENHANCE** UP and DOWN (top and bottom line of the figure), which represent situations in which a genomic aberration is mirrored by a translational regulation in the same direction (i.e., a gene which is deleted and also downregulated at the translatome level, thus magnifying the alteration induced by the CNA; or vice versa), and **RESTORE UP** and DOWN (second and third line of the figure), in which a genomic aberration is potentially compensated for by a translational regulation in the opposing direction (i.e., a gene which is gained but downregulated at the translatome level, thus limiting the effects of the genomic aberration; or vice versa).

Allelic gains and losses due to cancer genomic instability can have a direct effect on the affected loci and a more unpredictable, indirect effect on other loci due to the extensive networks linking gene products in cells. To understand how translational control can impact the neuroblastoma phenotype, we considered the simpler case of the direct, collinear effects of gene dosage changes on the translational efficiency of the gene itself. From the CNAs mapped to the 13 MYCN-amplified neuroblastoma cell lines, we extracted those impacting the loci of differentially represented genes. We identified 157 differentially represented genes (12.2% of the total) bearing collinear CNAs, 51 of which were deletions and 106 of which were gains. Among these, we observed a “compensatory” translational efficiency for 92 genes: 23 highly expressed genes carrying a genomic deletion (positive compensation) and 69 poorly translated genes with a genomic gain (negative compensation). We termed these genes “RESTORE”, as they compensate for genomic disruptions by restoring the normal levels of their cognate proteins. RESTORE UP genes are translationally enhanced to compensate for a genomic loss, while RESTORE DOWN genes follow the opposite pattern. Among the remaining 65 genes, 37 had gained copies and were efficiently translated, whereas 28 were partially deleted and inefficiently translated: these are “ENHANCE” genes, as their translational behavior magnifies the genomic alteration effect on gene expression. ENHANCE UP genes are positively magnified (gained and highly translated), whereas ENHANCE DOWN genes follow the opposite pattern. These behaviors are depicted in Fig. 3, with the corresponding genes listed in Supplementary Table S2.

**Figure 4:**
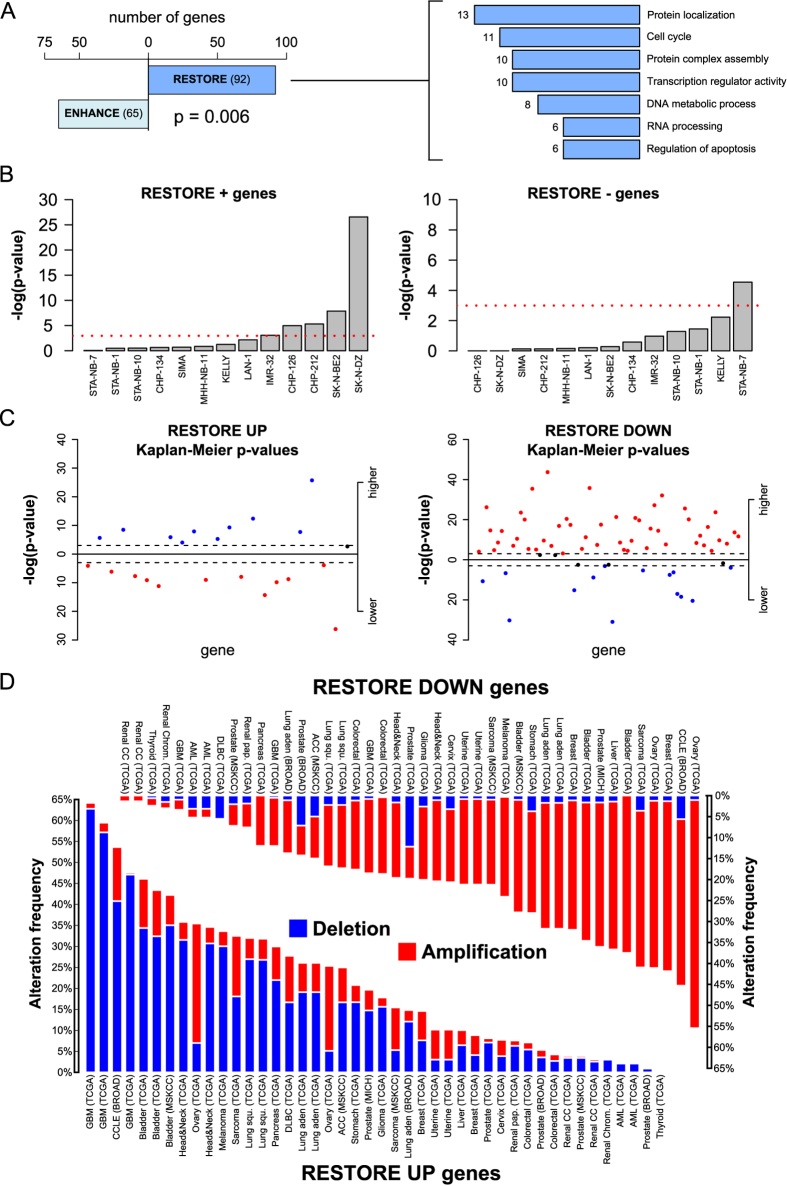
RESTORE genes are biologically relevant. (**A**) The proportion of RESTORE and ENHANCE genes in differentially represented genes (left), which is significantly higher for the former (p-value 0.006). The right panel shows the most represented biological processes and molecular functions for RESTORE genes, with their number of genes indicated next to the bar. (**B**) p-values for the global prevalence of RESTORE UP (left) and RESTORE DOWN (right) patterns in single cell lines. The red dotted line indicates the significance threshold at 0.05. (**C**) The −log10 p-value for RESTORE genes is associated with a worse neuroblastoma prognosis when their expression is higher (upper part) or lower (lower part). Red dots represent genes whose association with prognosis is in accordance with the RESTORE behavior (higher expression for RESTORE DOWN, lower for RESTORE UP), and blue dots represent genes with the opposite behavior. Dashed lines around the zero line represent the 0.05 p-value threshold. (**D**) The overall frequency of genomic alteration in several tumor types for the neuroblastoma RESTORE UP genes (lower part) and RESTORE DOWN (higher part). These genes almost entirely undergo concordant alterations in most tumor types, with RESTORE UP being mostly deleted and RESTORE DOWN being mostly gained.

We then focused on the RESTORE genes, given their potential to be involved in the fitness of tumor cell clones by counteracting genomic imbalances. To determine whether this potential is indeed realized in neuroblastoma, we predicted and verified three possible features of these genes. First, the RESTORE behavior should be more frequent than that of ENHANCE because RESTORE behavior counteracts gene expression imbalances that derive from the many segmental alterations observed in neuroblastoma cells. As expected, RESTORE events are significantly more prevalent than ENHANCE events (binomial test p = 0.006), which is a reflection of the cell lines’ tendency to buffer DNA imbalances with mRNA translational compensation (Fig. 4A, left). Furthermore, genes with compensatory translational efficiency found in CNAs (thus our RESTORE genes) are enriched with respect to all genes with compensatory translational efficiency in the genome (Fisher test p = 5.01E-09). Moreover, this behavior may be generalizable to CNA genes in individual lines, not just the differentially represented genes we identified. Therefore, we extracted the gained and lost genes for each cell line, coupled to their translational efficiency distributions, and computed Wilcoxon test p-values under the hypothesis that we should observe higher translational efficiencies for lost genes and lower translational efficiencies for gained genes with respect to genes not altered in copy number. Five cell lines (CHP-126 and CHP-212, IMR-32, SK-N-BE2 and SK-N-DZ) have a significant p-values for RESTORE UP compensation and one (STA-NB-7) for RESTORE DOWN (Fig. 4B). Considering all the cell lines, we also found that the RESTORE UP p-value strength was correlated with the translational efficiency distribution breadth (Pearson r = 0.59), suggesting that the occurrence of the RESTORE pattern may be more widespread in cell lines subjected to stronger translational control. Therefore, the RESTORE behavior could compensate for fitness drops due to the presence of segmental alterations in neuroblastoma clones, thus substantially contributing to tumor onset and progression by enhancing cell viability.

Second, we would expect RESTORE genes to be associated with neuroblastoma aggressiveness. RESTORE genes do not considerably overlap with classic cancer genes: indeed, as listed in Supplementary Table S3, intersecting RESTORE genes with TUSON cancer gene predictions^18^ yields only 16 of the 92 predicted to be potential oncogenes or tumor suppressors. Further annotations, listed in the same table, show very little overlap with pan-cancer copy-number alteration drivers (1/92 genes)^19^ or stemness determinants^20^ (3/92 genes) and only a moderate enrichment of cell essential genes (13/92, Fisher’s test p = 1.45E-05) defined by RNAi screenings in HeLa cells^21^,^22^,^23^. RESTORE genes might therefore be a novel group of genes related to cancer biology. We thus studied their association with prognosis using Kaplan-Meier curves constructed from the transcriptomic profiles of publicly available neuroblastomas^24^. As shown in Fig. 4C and listed in Supplementary Table S4, RESTORE DOWN genes are predominantly associated with a worse prognosis when highly expressed (48/69 have a significant p-value), whereas 10 of 23 RESTORE UP genes are significantly associated with a worse prognosis when poorly expressed. Globally, 63% of RESTORE genes are significantly associated with neuroblastoma prognosis when in the expression status that would trigger the translational compensation observed in our cell lines.

Third, the ability of RESTORE genes to undergo CNAs that may be translationally compensated to reestablish fitness could be independent of the tumor type; we would thus expect these genes to bear the same CNAs in other tumors, as the degree of freedom granted by this compensation could favor the “fixation” of these CNAs during clonal evolution. To test this possibility, we studied the occurrence of CNAs involving RESTORE genes across 27 tumor types using the cBio portal^25^. Indeed, RESTORE genes predominantly display the same kind of genomic lesions in the majority of tumor types, with RESTORE UP genes being deleted (Fig. 4D, down) and RESTORE DOWN genes being gained (Fig. 4D, up). A 1000-sample bootstrap of randomly selected genes indicated that both deletion (RESTORE UP) and gain (RESTORE DOWN) frequencies are significantly higher than expected (p < 0.001). When performed on randomly selected genes from regions of genomic alteration in neuroblastoma (excluding RESTORE genes), the frequency of deletions (RESTORE UP loci) is significantly higher than expected (p < 0.001), whereas the frequency of gains (RESTORE DOWN) is not (p = 0.592).

Finally, we searched for factors that could play a role in translational compensation by analyzing the untranslated regions (UTRs) of the RESTORE gene mRNAs for experimentally determined cis-elements and RBP/miRNA-binding sites using the AURA 2^26^ database. The regulatory enrichment tool of AURA 2 retrieves experimentally annotated binding sites or cis-elements from the UTRs of specified mRNAs and computes an enrichment p-value for each RBP/miRNA/cis-element type found in at least one of these UTRs. To increase the likelihood of these trans-factors being related to the change in translational efficiency of the RESTORE genes, we selected only RBPs found in the CNA loci and processed our miRome profiles to select only the expressed miRNAs from such loci. The resulting candidates of most interest are trans-factors with potential multiple interactions: the PUM2, LIN28A and FMR1 RBPs (associated with 28, 37 and 43 mRNAs, respectively) and the miR-21, miR-106b and miR-301a miRNAs (associated with 4 mRNAs each) (Supplementary Fig. S3). For cis elements, the RESTORE DOWN mRNAs were observed to be enriched in alternative polyadenylation sites (39 genes, 56.5%, Fisher’s test corrected p < 0.001) and AU-rich elements (44 genes, 63.7%, Fisher’s test corrected p < 0.001); no significant enrichment was detected in the RESTORE UP genes.

### Translational restoration of histone complex stoichiometry

**Figure 5:**
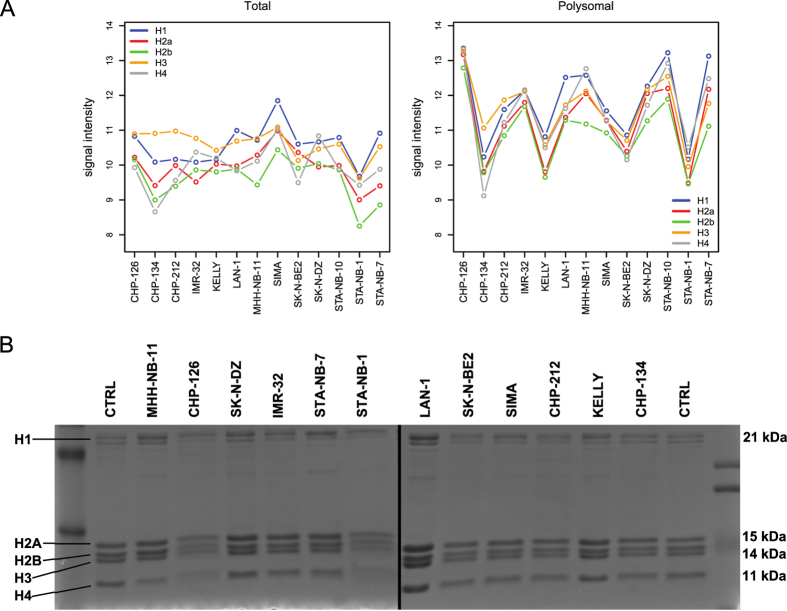
A translational compensatory mechanism for coordinated histone gene expression. (**A**) Expression levels for the upregulated histone genes in all the cell lines averaged by histone family at the transcriptomic (left) and at the translatomic (right) levels. Each color identifies a different histone family, and the cell lines are represented on the horizontal axis. Blue rectangles highlight the almost absent compensation of the KELLY cell line. (**B**) Coomassie staining of the histone protein extracts of the 13 MYCN-amplified neuroblastoma cell lines we analyzed, highlighting the restoration of a proper stoichiometry between the various histone types. CTRL lanes contain a commercial histone extract at a known concentration.

RESTORE genes were defined as those genes that are localized to copy-number-altered regions and that possess mRNAs differentially abundant between the transcriptome and the translatome, thus selecting for those loci that translational regulation may directly affect to compensate for DNA imbalances. However, as already suggested in Fig. 4A,B, this definition forced us to select a small sample of all genes with altered translation rates, the majority of which are instead likely indirectly affected. To validate examples of such genes, we returned to the differentially represented genes reported in Fig. 2B, whether they were encoded in unbalanced loci. The most enriched class contained histone genes, which nonetheless are not perturbed by CNAs (Supplementary Table S1). Given their importance for cell survival and proliferation^27^, the rigid stoichiometric arrangement of their protein products in the nucleosome and the known mechanism by which they are translationally controlled^28^, we employed them to test the generality of the RESTORE behavior in neuroblastomas. We first reported their expression in the cell lines; Supplementary Figure S4 displays a sample of all the scored histone genes in a single cell line and of a single gene in all cell lines. These genes include members of all replication-dependent histone families (3 genes for H1, 12 for H2A, 16 for H2B, 5 for H3 and 9 for H4). The nucleosome relies on the tight stoichiometry of its components to be functional^29^,^30^. Because histone type variants are highly similar in sequence and associate to produce a single component of this complex (e.g., all H4 variants produce H4 proteins), we reasoned that the mRNA signals of the histone genes should be grouped by family. We thus averaged the sum of histone mRNA levels by family, both in the transcriptome and the translatome. The results shown in Fig. 5A show a coordination of translated histone mRNA levels that is absent in the transcriptome. We confirmed that protein levels correspond with the expected nucleosome stoichiometry by performing histone extraction followed by Coomassie staining (Fig. 5B). The microarray signals of one histone gene per type were also validated by RT-qPCR in four of the cell lines (Supplementary Fig. S4). The experimental validation of a clear translational restoration pattern for the histone genes in neuroblastoma cells may thus suggest that the tendency to reinstate “normal” expression levels by translational modulation is not limited to CNA-imbalanced genes. The occurrence of such transcriptome imbalances may thus be due to indirect effects, such as the genomic alteration of a transcriptional or post-transcriptional regulator.

Searching for regulators of histone mRNAs by means of AURA 2^26^, we eventually observed the well-known histone RNA hairpin-binding protein SLBP as potentially regulating at least 50% of these genes (p = 8.9E-10). Interestingly, one cell line (KELLY) appears almost unaffected by the translational restoration of stoichiometry (as visible in Fig. 5A). Investigating the genomic status of histone-associated factors in these cells, we found that SLBP is heterozygously deleted in KELLY and not in all the other cell lines. SLBP may thus be involved in the reduced compensatory pattern in KELLY cells.

Because the formation of fixed-stoichiometry protein complexes, such as the nucleosome, can be a strong determinant of functionality for core cell activities, we wanted to determine if the behavior demonstrated above is a more general feature of protein complexes. We thus retrieved the 1725 human protein complexes annotated in CORUM^31^: out of these, 126 (7.3%) contained half of the genes belonging to our differentially represented group. Globally, a significant number of differentially represented genes (208, 16.2%) are part of a complex (1000-sample bootstrap p = 0.005). The significant enrichment of genes coding for protein complex components among the differentially represented genes suggests that translational restoration may be particularly active in the homeostasis of protein complexes.

## Discussion

We performed an integrative analysis of thirteen neuroblastoma cell lines representative of high-risk neuroblastoma (Fig. 1A), in which we added translatomic profiles to the extant genome, transcriptome and miRome profiles. Although cancer-related gene catalogs do not score the RBP category significantly, we found a statistically significant enrichment of RBPs and miRNAs among the loci affected by CNAs in our cell lines that was greater than that of conventional cancer-related gene categories, such as transcription factors^32^ or protein kinases^33^. We thus hypothesized that genes involved in the post-transcriptional control of gene expression may be specifically affected by segmental aberrations in neuroblastoma. While the involvement of translational control in cancer has been widely explored in studies of mTORC1 pathway protein mutations^34^,^35^ and the altered expression of translation initiation complex components^36^,^37^, little is known about the role of RBPs. RBP involvement in neuroblastomas should therefore be further explored, especially given that LIN28B, an RBP involved in miRNA maturation^38^ and in translation^39^, has recently been identified as a determinant of inherited neuroblastoma predisposition^40^ and a powerful oncogene able to recapitulate this disease in mice^41^.

Our evaluation of tumor mRNA translational efficiencies is the first attempt of this type in patient-derived cells, preceded only by an analysis in glioma based on a murine model^10^ in which polysomes and non-translating mRNA-bound single ribosomes could not be distinguished. The use of a low-throughput method, sucrose-gradient centrifugation, on a sufficient number of cell lines representative of a single aggressive tumor subtype was our proof-of-principle demonstration of the usefulness of translatomic profiling in cancer studies.

We identified genes for which the differences in transcriptomic and translatomic signals were shared among cell lines and were therefore features of the corresponding tumor subtype. This study demonstrated the occurrence of active translational control by which transcriptomic fluctuations are reprogrammed at the translatome level. We identified the genes most affected by this feature. These differentially represented genes comprised more than 10% of the total (1288/11,321 detectable genes); of these, more than 10% were also located in sites of genomic alteration. For these genes, we hypothesized that their translational efficiencies were likely directly due to allelic imbalance. We focused on those displaying RESTORE behavior, the countering of allelic imbalance with compensatory translational efficiency, i.e., the more efficient translation of loci with lost alleles and the more inefficient translation of loci with gained alleles. We demonstrated that this pattern is not due to chance and is not restricted to these few genes. Given the relative paucity of point mutations^24^ and cancer-driving genes in neuroblastomas (less than a dozen established in almost twenty years of investigation, see Fig. 1A), the several segmental alterations of high-risk tumors may still hold undetected determinants of the tumor phenotype, as these alterations are often associated with prognosis^42^. However, considering the failure to discover the drivers of neuroblastoma tumors using conventional paradigms, other approaches should be explored.

In our proposed model, RESTORE genes suffer a lack or excess of expression due to a CNA negatively affecting the fitness of the clone in which they occur. A compensatory alteration then modulates their translation rate to promote restored protein levels and fitness; when this occurs, the tumor becomes immune to the allelic imbalance of these otherwise dose-sensitive genes. The observation that RESTORE genes have the same alteration in 27 other tumor types (Fig. 4D) suggests that this “dosage permissiveness” phenomenon is active in other cancer types. RESTORE genes, despite not being conventional cancer genes^43^, may behave as new, powerful drivers able to bypass the bottleneck of loss of clonality potential induced by dosage alteration through translational compensation. Such a role would justify the association of a considerable proportion of these genes with poor prognosis when altered in a manner that enables translational restoration (Fig. 4C).

We also showed that this behavior is not restricted to dosage effects induced by segmental alterations and could therefore involve a larger fraction of genes. Genomic instability indirectly affects the expression of many more genes than those located in CNAs, both in neuroblastoma^44^ and other tumors^45^, thus altering the levels of mRNAs in normally biallelic loci. These altered levels could also be “corrected” by changes in translational efficiency. We show here the paradigmatic case of histone mRNAs, for which a strong pressure towards cell proliferation restricts the stoichiometry of nucleosome protein components. The frequent involvement of translational control in assuring protein complex stoichiometry was observed in a recent work in bacteria^46^.

This homeostatic model of cancer progression by translational compensation may bring under scrutiny a number of new genes, not necessarily involved in cancer biological hallmarks, whose fluctuation in mRNA levels would simply suppress clonality unless a counteracting fluctuation in translational efficiency restores it. These “fitness bottleneck” genes could become new therapeutic targets, along with the translational mechanisms enabling their normalization.

Given this compensatory behavior, translational efficiency thus appears as a better indicator of prognostic relevance than transcriptomic profiling. However, one should consider that the translatome is a proxy for protein levels and not a direct measurement; other mechanisms may influence the final outcome of the translational compensation. A high-throughput proteomic profile would grant the closest observation platform for tumor phenotype; however, still unsolved issues prevent the generation of profiles that are as complete as those of the transcriptome and the translatome.

The present technical limitations of translatome profiling in cancer preclude studies with large cohorts of patients; however, this limitation could rapidly change with the development of new RNA-seq-based techniques stemming from ribosome profiling^47^, thus paving the way to systematically study and exploit the translational compensation of genomic instability in cancer.

## Materials and Methods

### Cell culture

Cell lines were grown according to the suppliers’ instructions at 37 °C in a 5%-CO_2_ humidified atmosphere. CHP-134, IMR-32, KELLY, LAN-1, SK-N-BE2 and −DZ were obtained from ECACC (Salisbury, UK). CHP-126, MHH-NB-11 and SIMA were obtained from DSMZ (Braunschweig, Germany). CHP-212 was obtained from ATCC. STA-NB-1, −7 and −10 were kindly provided by Dr. Peter F. Ambros (CCRI, Vienna, Austria). All cell lines were used at early passages (n = 3) to avoid the insurgence of any alteration, and all were checked for mycoplasma and other potential infections. All cell lines were checked against the Database of Cross-contaminated or Misidentified Cell Lines (http://iclac.org/), and none were found to have been previously flagged as cross-contaminated or misidentified.

### aCGH microarrays

Total DNA was isolated according to the manufacturer’s protocol using the DNA Blood and Tissue Extraction Kit (Qiagen). Array-CGH was performed using Human Genome CGH 244 K microarrays (Agilent Technologies), and the slides were scanned using a G2565BA scanner (Agilent Technologies).

### Total RNA profiling

Total RNA was isolated according to the manufacturer’s protocol using the RNeasy Mini Kit (Qiagen) and then quantified and quality-assessed using the RNA 6000 Nano Assay on the 2100 Bioanalyzer (Agilent Technologies); a 7 RIN threshold was used to select samples for this study. Expression profiling was performed with 500 ng of starting material. The samples were hybridized on Human GE 4x44K v2 microarrays (Agilent Technologies), and the slides were scanned using a G2565BA scanner (Agilent Technologies).

### Polysomal RNA profiling

Cells were incubated for 3 min with 0.01 mg/ml cycloheximide at 37 °C. Then, the plates were placed on ice, the medium was removed, and the cells were washed twice with PBS supplemented with 0.01 mg/ml cycloheximide. The cells were lysed with 300 μl of cold lysis buffer (10 mM MgCl_2_, 10 mM NaCl, 10 mM Tris–HCl (pH 7.5), 0.2 U/ml RNase inhibitor (Fermentas), 1 mM DTT, 1% Triton X-100, 1% sodium deoxycholate and 0.01 mg/ml cycloheximide) and scraped. The resulting extracts were centrifuged for 5 min at 12,000 g at 4 °C. The supernatant was loaded on a 15–50% linear sucrose gradient with 30 mM Tris–HCl (pH 7.5), 100 mM NaCl and 10 mM MgCl_2_ and centrifuged on an SW41 rotor for 100 min at 180,000 g. Fractions were collected by monitoring the absorbance at 254 nm and were treated with 0.1 mg/ml proteinase K for 2 h at 37 °C. RNA was extracted with phenol–chloroform, precipitated with isopropanol and resuspended in 30 μl RNase-free water. All fractions after the ribosomal 80S peak were considered as polysomal and employed for these analyses. Expression profiling was performed as described for total RNA.

### miRNA profiling

miRNAs were isolated according to the manufacturer’s protocol using the miRNeasy Micro Kit (Qiagen) and then quantified and quality-assessed using the Small RNA Assay on the 2100 Bioanalyzer (Agilent Technologies); a 7 RIN threshold was used to select samples for this study. miRNA profiling was performed with 100 ng of starting material. The samples were hybridized on Human miRNA Microarrays 2.0 (Agilent Technologies), and the slides were scanned using a G2565BA scanner (Agilent Technologies).

### Histone extraction

Histone proteins were purified from nuclear pellets by acid extraction. Cells were resuspended in the extraction buffer (PBS containing 0.5% Triton X-100, 2 mM PMSF, and 0.02% NaN_3_) and incubated on ice for 10 min. The pellet was collected by centrifugation at 10,000 × *g* for 10 min at 4 °C, washed once in PBS containing 0.5% Triton X-100 and resuspended in 400 μl of 0.2 M HCl. After 4 h of incubation on ice, the supernatant was collected by centrifugation at 14,000 × *g* for 15 min, and the proteins were recovered by cold acetone precipitation.

### Coomassie staining

Histone proteins were separated by a 15% SDS–PAGE assay. The gel was stained with Coomassie Brilliant Blue R-250, destained in 40% methanol and 10% glacial acetic acid and visualized using the ChemiDoc XRS + Imaging System (Bio-Rad). A commercially available calf thymus histone preparation (Roche) was used as a positive control.

### qPCRs

qPCRs were performed using TaqMan probes (Applied Biosystems). cDNA was synthesized from 1 μg of RNA in 20-μl reactions using the iScript cDNA Synthesis Kit (BioRad). PCR mixtures contained 10 ng of cDNA; the mix was prepared using 2× Kapa Probe Fast qPCR Universal Master Mix (Kapa Biosystems) and 1x TaqMan probe. PCR reactions were performed in triplicate on a CFX96 real-time PCR system (BioRad). The cycling conditions were 3 min at 95 °C and 40 cycles at 95 °C for 30 sec, 60 °C for 20 sec and 72 °C for 60 sec. mRNA levels were computed by the delta-CT method using the geometric means of HPRT1, B2M and SDHA for normalization.

### aCGH data analysis

Arrays were loaded in R by limma^48^, the signals were median-centered, and MANOR^49^ was used to correct for global intensity trend and local spatial bias and to check the signal-to-noise ratio and replicate consistency. Filtered probes were processed with CGHcall^50^, applying an outlier smoothing correction, CBS segmentation and a post-segmentation normalization to adapt the data to its most likely zero-value. Genomic segments were assigned a loss/gain status, and recurrent aberrations were obtained through KCsmart^51^. RESTORE gene CNAs were analyzed in other tumor types with CGDS-R^25^. Primary high-risk neuroblastomas were retrieved from GEO^52^ (accession number GSE45478) and processed as described above. Alterations from both datasets were plotted with Circos^53^.

### Gene expression data analysis

Arrays were loaded in R through Agi4x44PreProcess (bioconductor.org/packages/2.13/bioc/html/Agi4x44PreProcess.html). Probes were filtered with the following criteria: at least 25% of the samples having values well above background; at least 75% having sufficient spot diameter and a signal-to-noise ratio well above the negative controls; and not saturated or an outlier. Probes were median-summarized and log2-converted; quantile normalization was applied through the same package. Genes with expression levels below the first quartile were considered non-expressed. Hierarchical clustering was performed using the hclust function (ward linkage, Pearson distance). The PCA analysis was performed using the prcomp function and k-means clustering using the kmeans function (with n = 3). Differentially represented genes were computed by RankProd^16^, SAM (cran.r-project.org/web/packages/samr) and T-test^54^. The corrected p-value threshold was 0.05 for T-test, PFP at 0.05 for RankProd and FDR at 0.05 for SAM.

### Gene annotations

Functional enrichments were performed with DAVID^55^, and cis-elements were identified using the AURA 2 regulatory element enrichment tool^26^. Essential genes were first obtained by merging three genome-wide siRNA screens in HeLa^21^,^22^,^23^ and intersecting our gene set with this list. Kaplan-Meier curves were computed for the RESTORE genes through R2 (http://r2.amc.nl). Human protein complexes were obtained from CORUM^31^.

### miRNA data analysis

Arrays were loaded into R by AgiMicroRna^56^. Probes were summarized and filtered for being expressed (at least 75% of the samples) and being well above negative controls (at least 25% of the samples). Signals were log2-converted, and quantile normalization was applied. Ingenuity Pathway Analysis (www.ingenuity.com) was used to perform functional enrichments on the miRNA list.

### RBP list construction

The RBP list was constructed by retrieving proteins annotated to contain an RNA-binding domain from InterPro^57^ and completed by adding novel RBPs identified by a recent work^58^.

## Additional Information

**Accession codes:** The microarray data were deposited in GEO^52^ under the accession number GSE56656.

**How to cite this article**: Dassi, E. *et al.* Translational compensation of genomic instability in neuroblastoma. *Sci. Rep.* **5**, 14364; doi: 10.1038/srep14364 (2015).

## Supplementary Material

Supplementary Information Supplementary Figures 1-4

Supplementary Information Supplementary Tables S1, S2A, S2B, S3, S4
