# Supplementary material for: Translational compensation of genomic instability in neuroblastoma
Source: Sci Rep. 2015 Sep 24;5:14364. doi: 10.1038/srep14364 (PMC4585852; doi:10.1038/srep14364)

Supplementary Information

Translational compensation of genomic instability in neuroblastoma

Erik Dassi[1], Valentina Greco[1], Viktoryia Sidarovich[1], Paola Zuccotti[1], Natalia Arseni[1], Paola Scaruffi[2], Gian Paolo Tonini[3] and Alessandro Quattrone[1,*]

Supplementary Figure S1: Clustering of transcriptomic and translatomic profiles. A) Principal component analysis of the profiles (first two components on x and y axis). Transcriptomic profiles are shown by a red square and translatomic profiles by a blue circle. **B)** Clusters identified by a k-means analysis (n=3) on the transcriptomic (red squares) and translatomic profiles (blue circles).

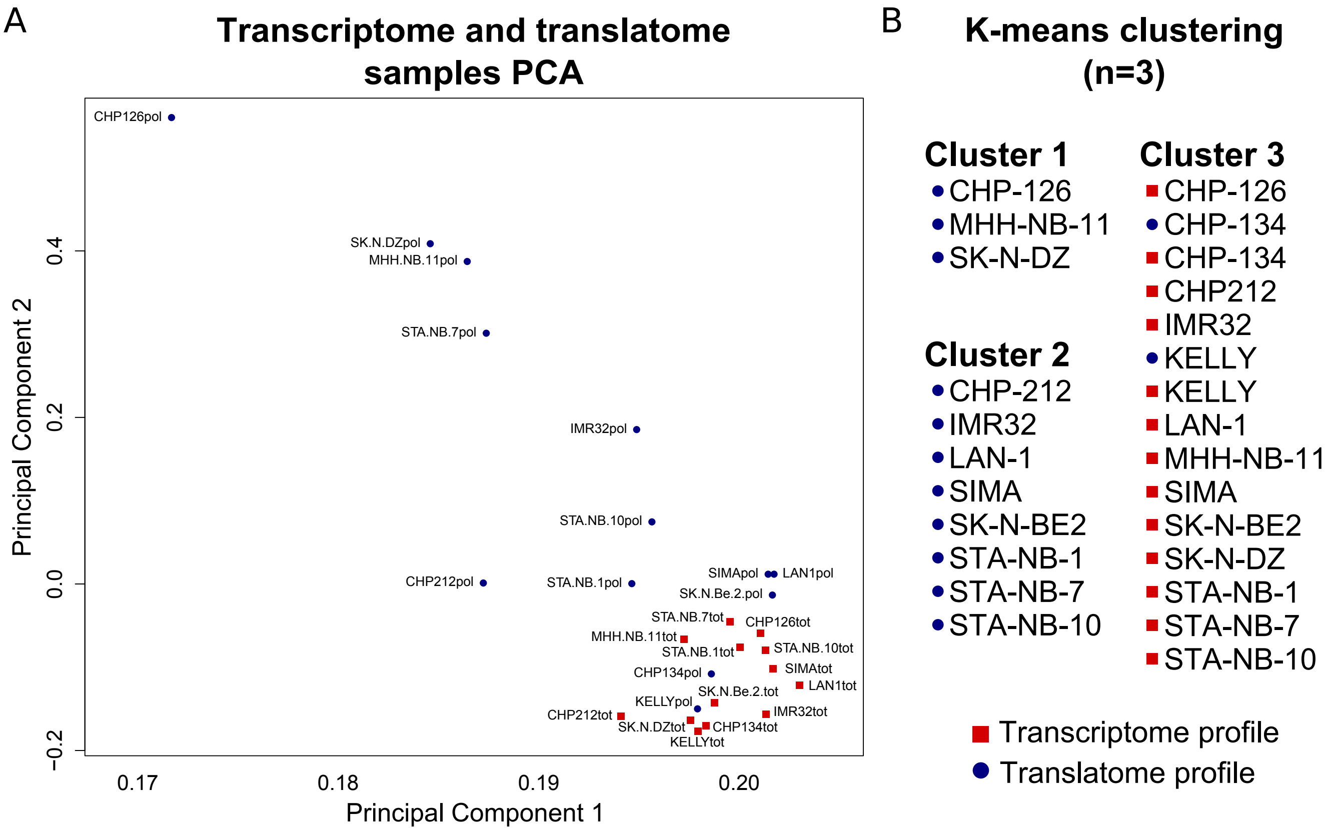

Supplementary Figure S2: Transcriptome versus translatome scatterplot. Scatterplots of transcriptomic (x axis) versus translatomic (y axis) microarray signal intensity data are shown for each of the 13 MYCN-amplified neuroblastoma cell lines employed for translatomic profiling.

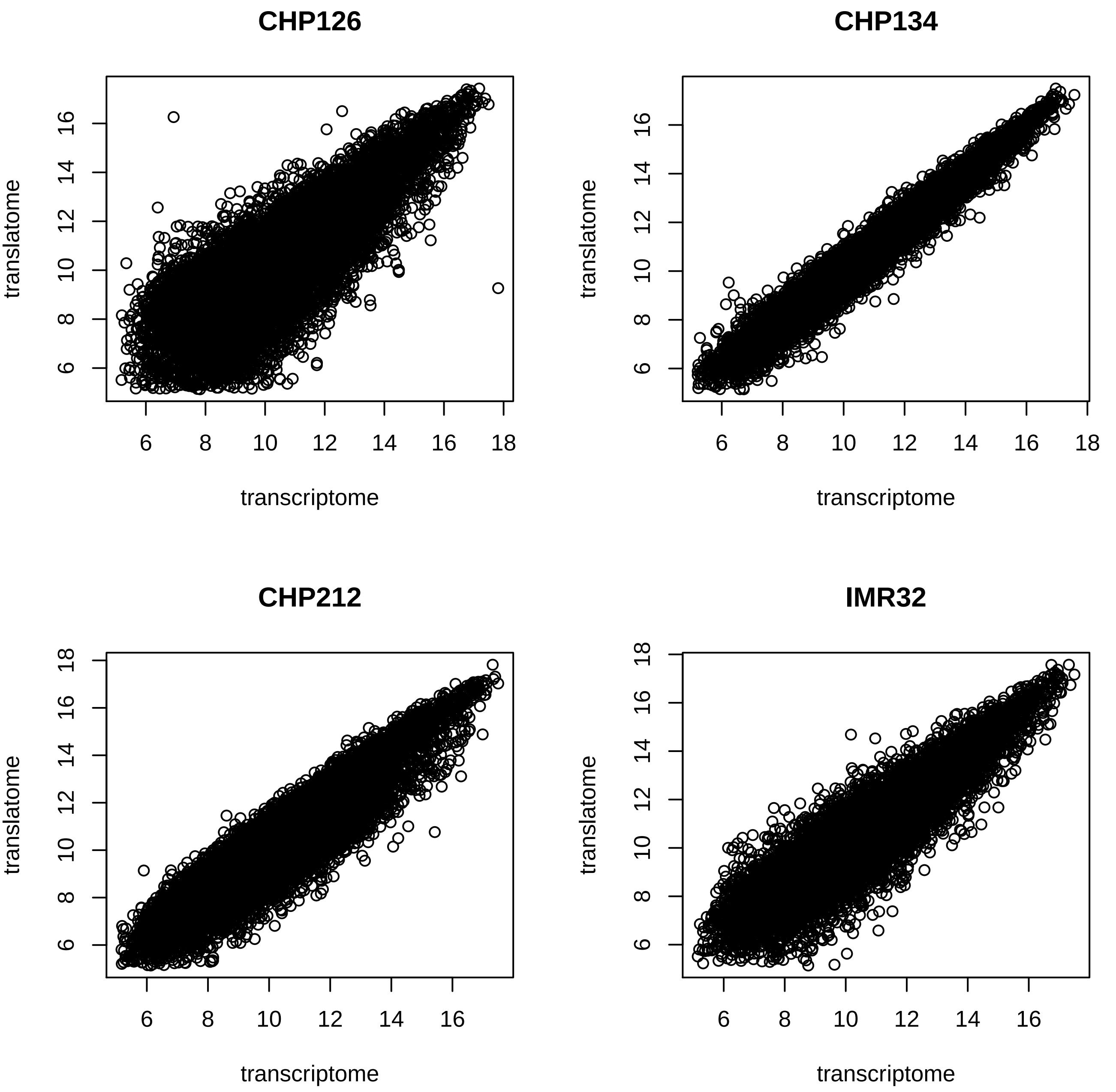
CHP126
CHP134
CHP212
IMR32
translatome
transcriptome
6
8
10
12
14
16
18

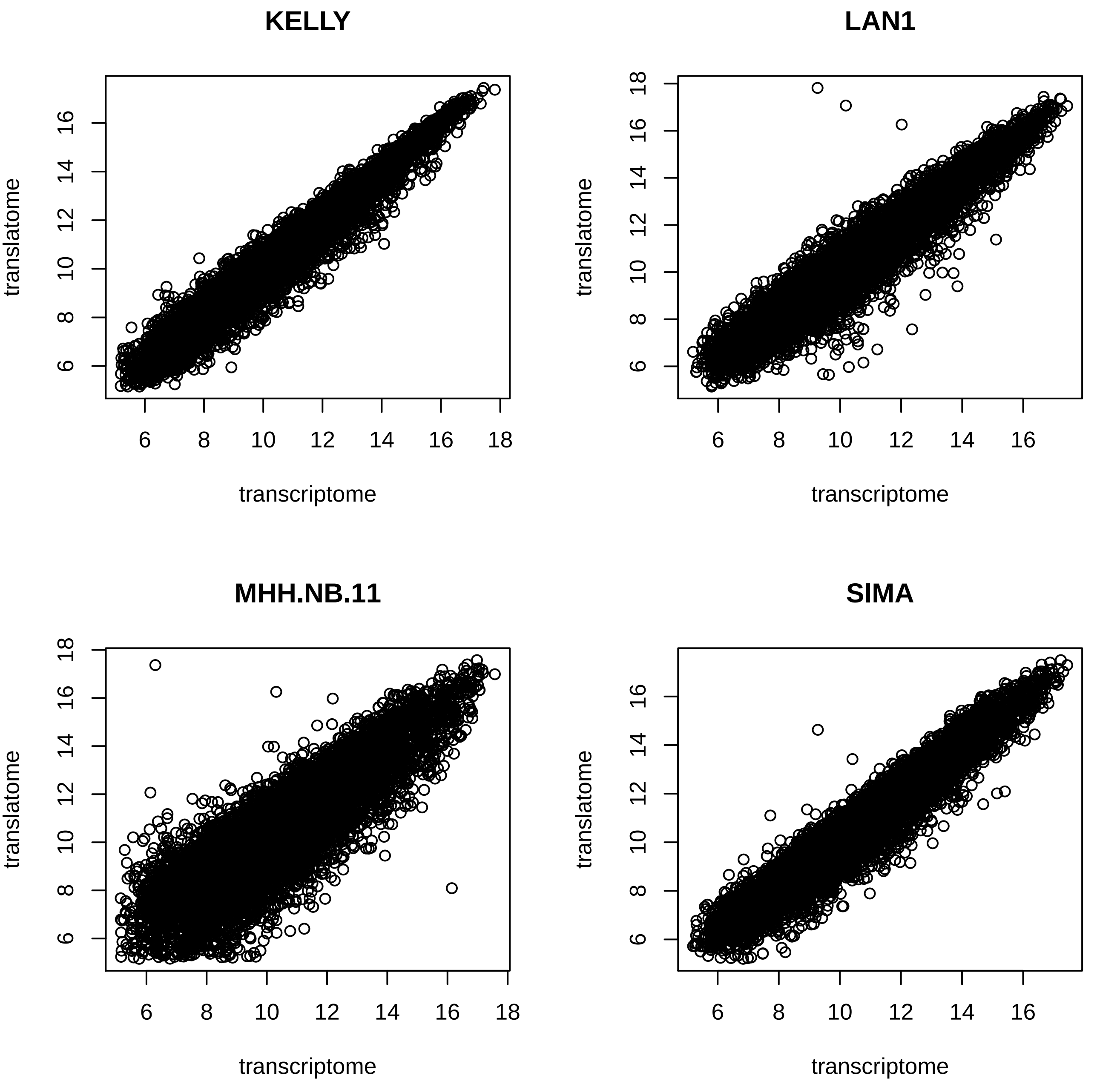

KELLY
LAN1
MHH.NB.11
SIMA
translatome
transcriptome
6
8
10
12
14
16
18

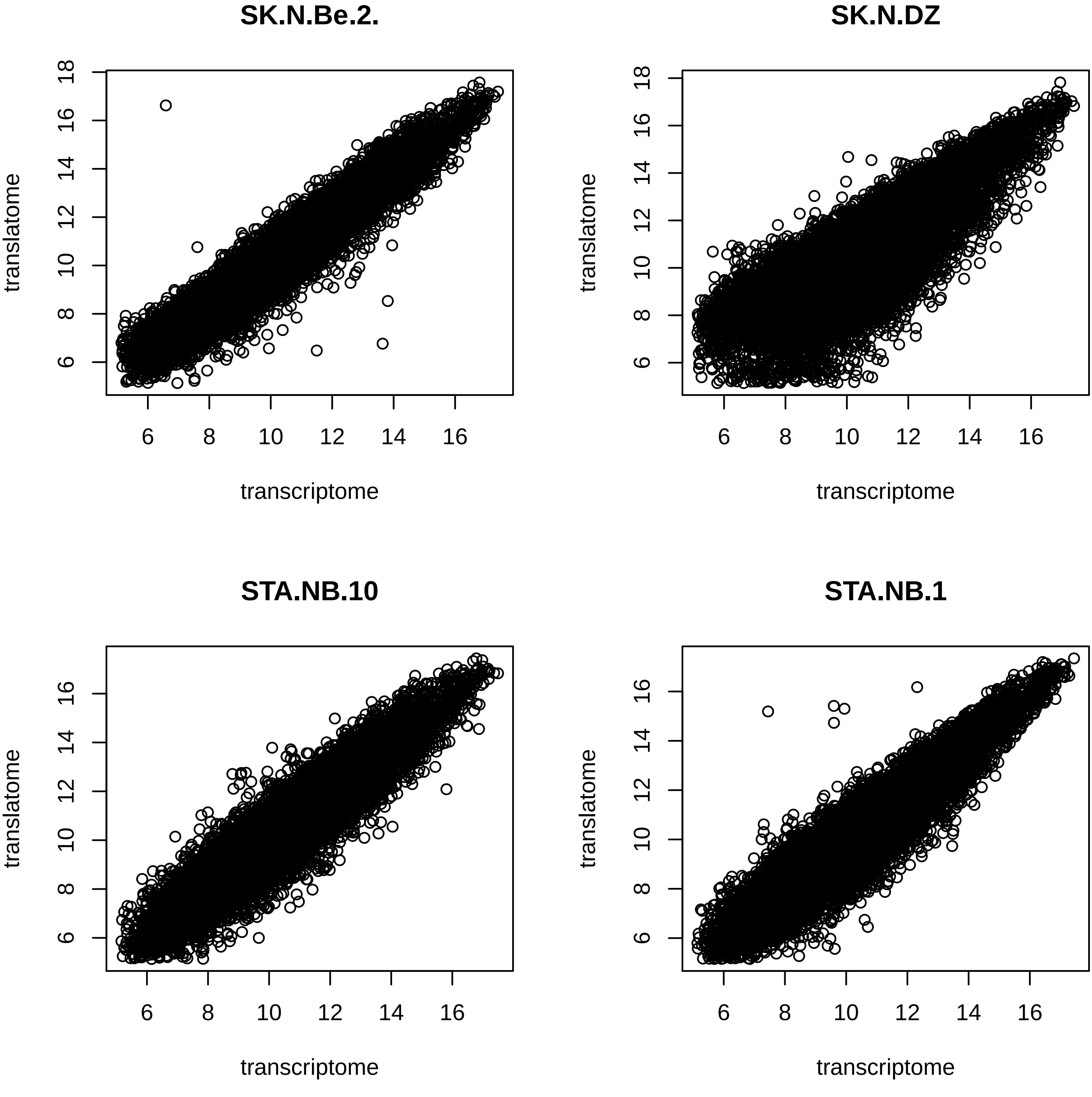
SK.N.Be.2.
SK.N.DZ
STA.NB.10
STA.NB.1
translatome
transcriptome
6 8 10 12 14 16 18
6 8 10 12 14 16

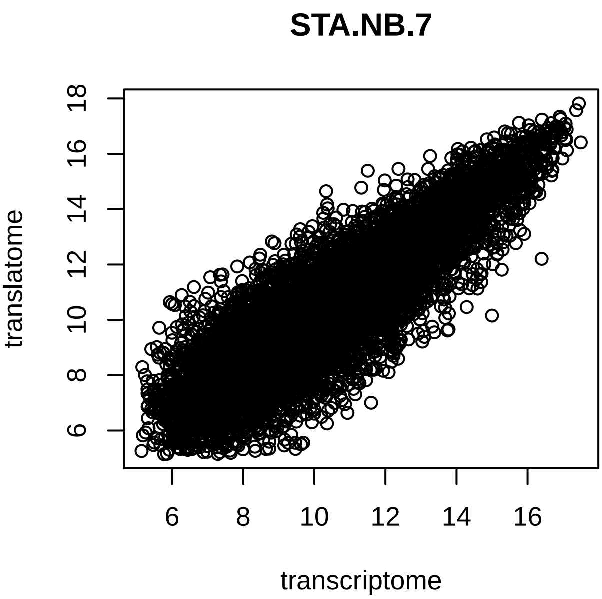

STA.NB.7
translatome
transcriptome
6
8
10
12
14
16
18

Supplementary Figure S3: Translational restoration by trans-factors. The network of altered RBPs, miRNAs and RESTORE genes in neuroblastoma cell lines is shown. Square nodes are RESTORE genes, and circular nodes are altered trans-factors (red indicates genomic gain, and blue indicates deletion). **A)** The network for RESTORE UP genes. **B)** The network for RESTORE DOWN genes.

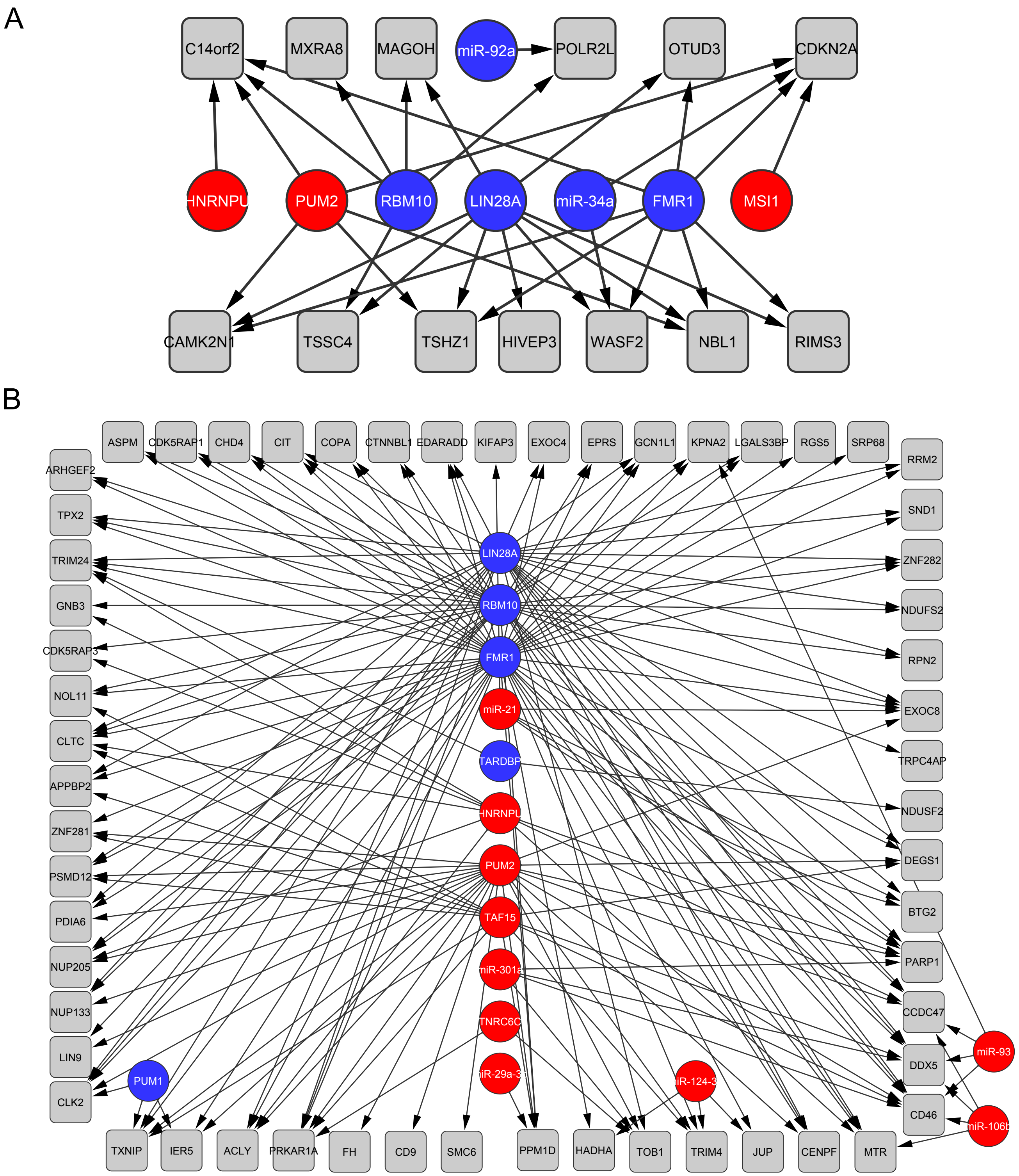

Supplementary Figure S4: Histone translational buffering in neuroblastoma at the single gene and single cell line level. A) Expression patterns at the transcriptomic (red) and translatomic (blue) levels for all histone genes involved in one MYCN-amplified neuroblastoma cell line. **B)** Expression pattern at the transcriptomic (red) and translatomic (blue) levels for one histone gene of the H3 family in all the MYCN-amplified neuroblastoma cell lines. **C)** All histone genes upregulated at the translatomic level, broken down by histone type. **D)** qPCR validation of 5 histone genes (one for each histone type) from four MYCN-amplified neuroblastoma cell lines. The plot represents qPCR fold change (in blue, computed as polysomal/total mRNA signal) versus microarray fold change (in red, computed as polysomal/total mRNA signal).

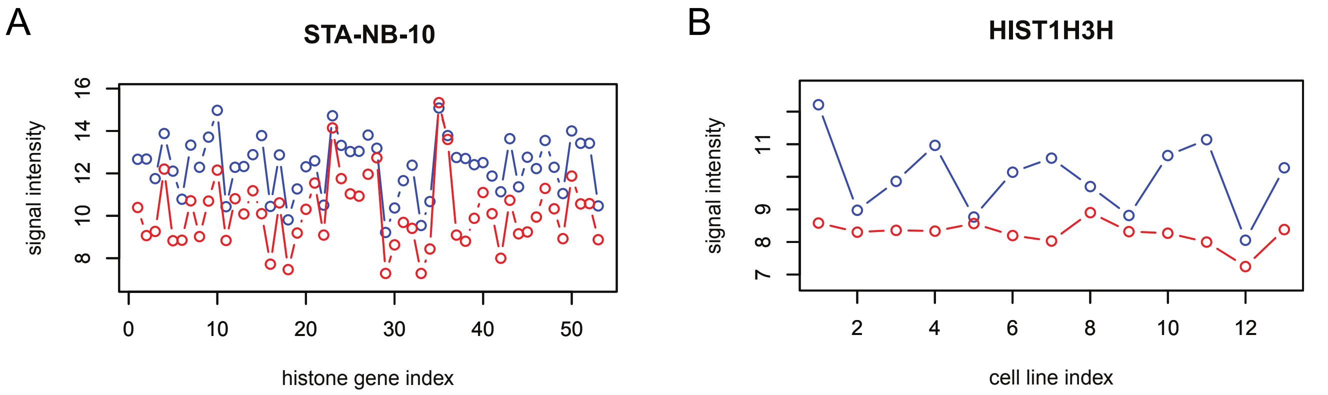

C

H1	H2A	H2B	H3	H4
HIST1H1C	HIST1H2AB	HIST1H2BB	HIST1H3B	HIST1H4B
HIST1H1D	HIST1H2AD	HIST1H2BC	HIST1H3D	HIST1H4D
HIST1H1E	HIST1H2AG	HIST1H2BD	HIST1H3F	HIST1H4E
	HIST1H2AH	HIST1H2BE	HIST1H3H	HIST1H4F
	HIST1H2AJ	HIST1H2BF	HIST1H3J	HIST1H4H
	HIST1H2AK	HIST1H2BG		HIST1H4I
	HIST1H2AM	HIST1H2BH		HIST1H4J
	HIST2H2AA3	HIST1H2BI		HIST1H4K
	HIST2H2AB	HIST1H2BJ		HIST2H4A
	HIST2H2AC	HIST1H2BK		
	HIST3H2A	HIST1H2BL		
	H2AFJ	HIST1H2BM		
		HIST1H2BN		
		HIST1H2BO		
		HIST3H2BB		
		H2BFS		

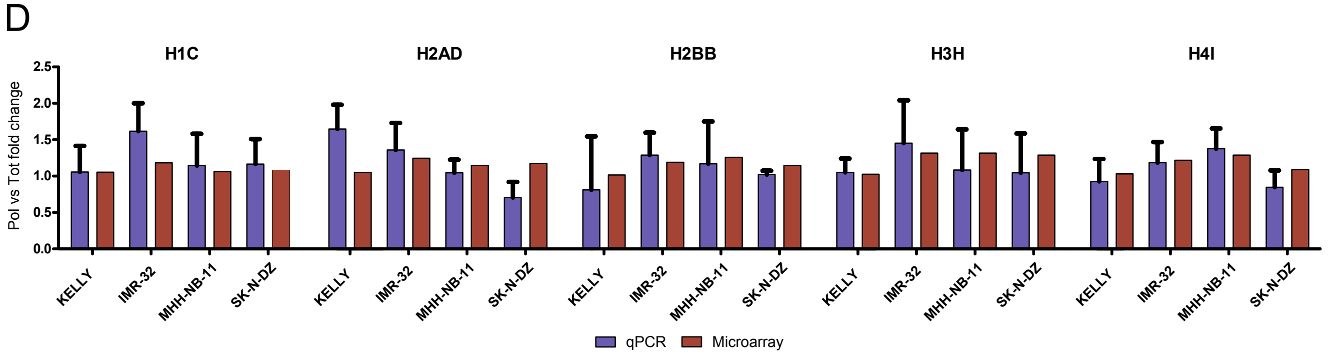

Supplement: Supplementary Information — Supplementary Figures 1-4 [file srep14364-s1.pdf]
